# Supplementary figures and images for: Identification of antibody-drug conjugate payloads which are substrates of ATP-binding cassette drug efflux transporters
Source: bioRxiv. 2025 Jul 18:2025.05.22.651305. Originally published 2025 May 27. Preprint. [Version 2] doi: 10.1101/2025.05.22.651305 (PMC12154920; doi:10.1101/2025.05.22.651305)

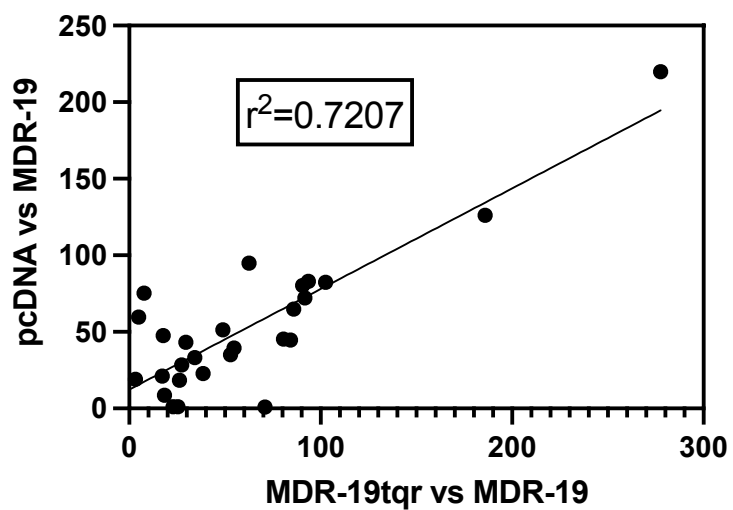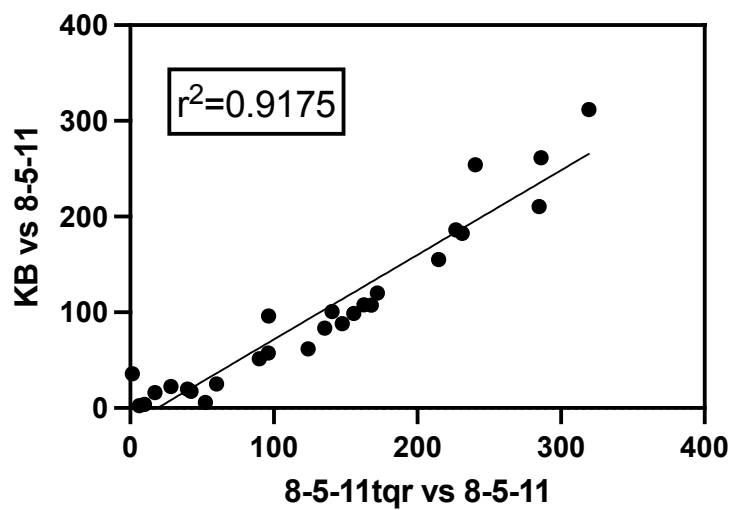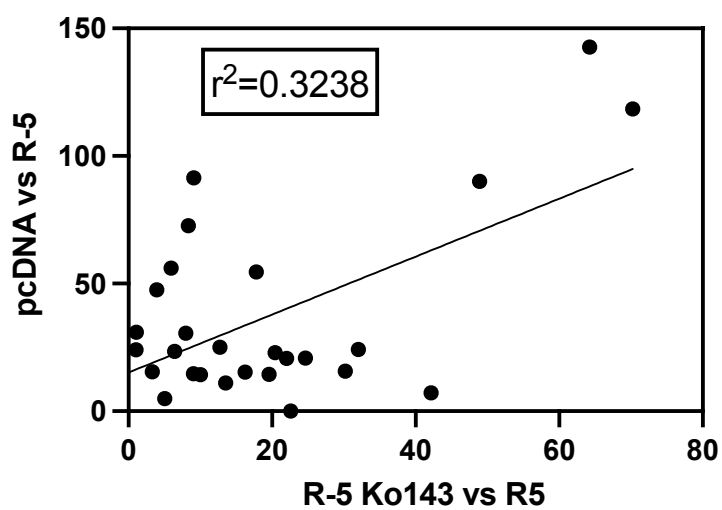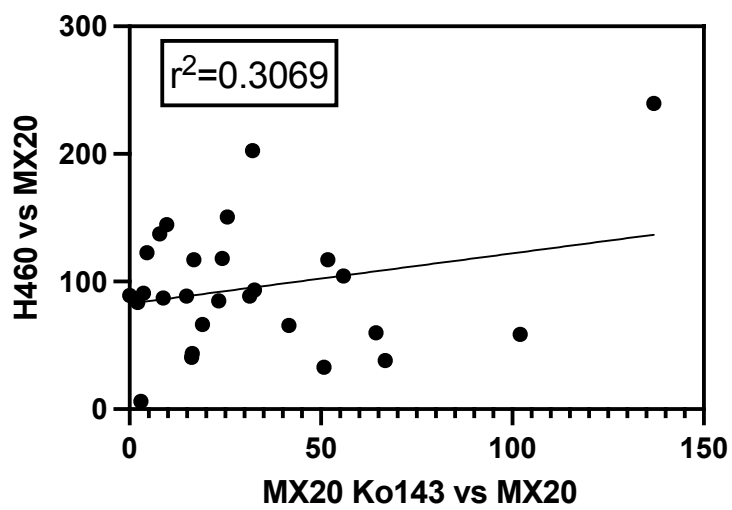

Supplement: Supplement 1 [file media-1.pdf]
