# Supplementary material for: Identification of antibody-drug conjugate payloads which are substrates of ATP-binding cassette drug efflux transporters
Source: bioRxiv. 2025 May 27:2025.05.22.651305. Preprint. [Version 1] doi: 10.1101/2025.05.22.651305 (PMC12154920; doi:10.1101/2025.05.22.651305)
Supplement: Supplementary file 1 [file NIHPP2025.05.22.651305v1-supplement-1.pdf]

bioRxiv preprint doi: https://doi.org/10.1101/2025.05.22.651305; this version posted May 27, 2025. The copyright holder for this preprint (which was not certified by peer review) is the author/funder. This article is a US Government work. It is not subject to copyright under 17 USC 105 and is also made available for use under a CC0 license.

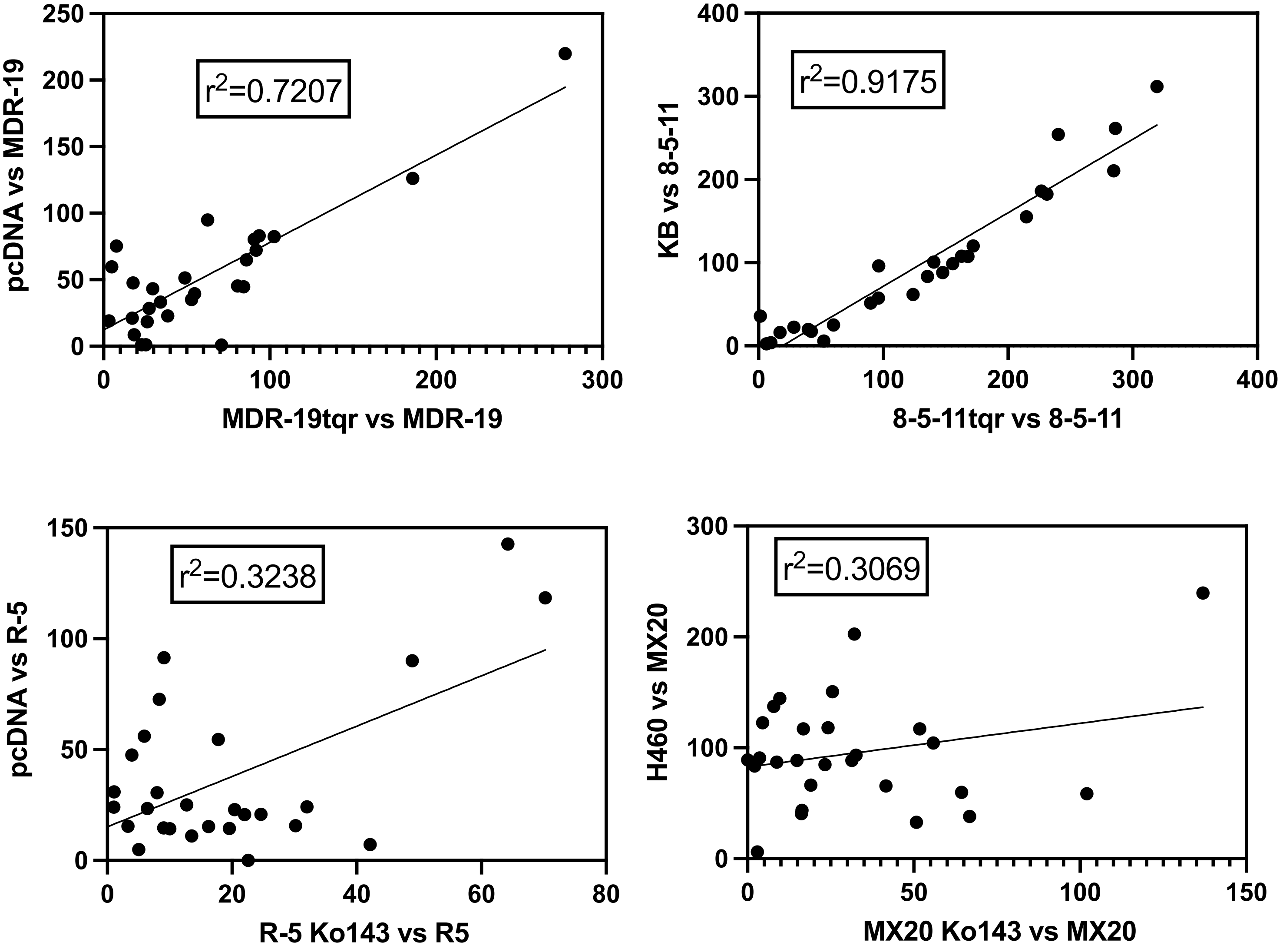

Supplementary Figure 1
